# Ultrasensitive Silicon Nanowire Biosensor with Modulated Threshold Voltages and Ultra-Small Diameter for Early Kidney Failure Biomarker Cystatin C

**DOI:** 10.3390/bios13060645

**Published:** 2023-06-13

**Authors:** Jiawei Hu, Yinglu Li, Xufang Zhang, Yanrong Wang, Jing Zhang, Jiang Yan, Junjie Li, Zhaohao Zhang, Huaxiang Yin, Qianhui Wei, Qifeng Jiang, Shuhua Wei, Qingzhu Zhang

**Affiliations:** 1 School of Information Science and Technology, North China University of Technology, Beijing 100144, China; hujiawei@ime.ac.cn (J.H.); liyinglu@ime.ac.cn (Y.L.); zhangxufang@ncut.edu.cn (X.Z.); wangyanrong@ncut.edu.cn (Y.W.); zhangj@ncut.edu.cn (J.Z.);; 2 Advanced Integrated Circuits R&D Center, Institute of Microelectronic of the Chinese Academy of Sciences, Beijing 100029, China; lijunjie@ime.ac.cn (J.L.); zhangzhaohao@ime.ac.cn (Z.Z.);; 3 State Key Laboratory of Advanced Materials for Smart Sensing, General Research Institute for Nonferrous Metals, Beijing 101402, China; weiqianhui@grinm.com

**Keywords:** silicon nanowire (SiNW), field effect transistor (FET), biosensor, cystatin C (Cys-C), acute kidney injury (AKI)

## Abstract

Acute kidney injury (AKI) is a frequently occurring severe disease with high mortality. Cystatin C (Cys-C), as a biomarker of early kidney failure, can be used to detect and prevent acute renal injury. In this paper, a biosensor based on a silicon nanowire field-effect transistor (SiNW FET) was studied for the quantitative detection of Cys-C. Based on the spacer image transfer (SIT) processes and channel doping optimization for higher sensitivity, a wafer-scale, highly controllable SiNW FET was designed and fabricated with a 13.5 nm SiNW. In order to improve the specificity, Cys-C antibodies were modified on the oxide layer of the SiNW surface by oxygen plasma treatment and silanization. Furthermore, a polydimethylsiloxane (PDMS) microchannel was involved in improving the effectiveness and stability of detection. The experimental results show that the SiNW FET sensors realize the lower limit of detection (LOD) of 0.25 ag/mL and have a good linear correlation in the range of Cys-C concentration from 1 ag/mL to 10 pg/mL, exhibiting its great potential in the future real-time application.

## 1. Introduction

Recently research has shown that the global mortality rate related to acute kidney injury (AKI) has far exceeded the total rates caused by breast cancer, heart failure and diabetes, and the mortality rate has remained high in the past 50 years [1,2,3]. The etiology of AKI is complex; in some areas, infection and trauma dominate AKI, whereas hypovolemic shock, sepsis, drugs or invasive treatments are the major causes in other areas [4]. In recent years, COVID-19 has hit the world. Although respiratory failure and hypoxemia are the main manifestations of COVID-19, renal involvement is also common [5,6]. The available data prove that in the context of SARS-CoV-2 infection, AKI can develop through many potential pathophysiological pathways [5]. Considering that the pathogenic factor of AKI has been lurking in our lives and AKI is fatal, the prevention and early detection of AKI are crucial.

Human serum cystatin C (Cys-C) is a cysteine protease inhibitor, which is synthesized by human cells and exists in various body fluids, including blood [7,8]. It is filtered from the blood by the glomerulus and then metabolized in the proximal tubules [9]. It is considered an early marker of AKI related to glomerular filtration rate (GFR) [10,11,12]. Recent studies have revealed that serum Cys-C concentration is more stable and accurate in GFR estimation and is independent of gender, diet, and muscle mass [11]. Therefore, it is of great significance to develop a fast, simple and highly sensitive Cys-C detection method for the prevention and early detection of AKI.

Thus far, many methods have been developed for Cys-C detection, such as enzyme-linked immunosorbent assay [12,13] and turbidimetry [14]. However, due to the high cost, long time consumption and limited sensitivity, the use of these methods has been limited in daily clinical work [15]. With the purpose of rapid detection of Cys-C, extensive attempts have been made to develop biosensors and immunosensors for Cys-C. Yang et al. (2016) developed an electrochemical genetic sensor that uses nanogold and iron oxide to amplify the signal [16]. Although this work has a low detection limit, it is not suitable for clinical application as the steps to convert the signal for this sensor are too complex. Additionally, Mi et al. (2016) prepared a photoelectrochemical immunosensor based on a TiO_2_ nanotube [17]. However, it is difficult to miniaturize due to the participation of the optical system. Desai et al. (2018) prepared electrochemical biosensors based on carbon nanotubes [18]. Due to the difficulty of the mass production of carbon nanotube materials, the large-scale preparation of the biosensors is limited, and it is difficult to reduce the production cost. Zhao et al. (2019) prepared a graphene-based electrochemiluminescence biosensor [19]. Although its detection limit is as low as 0.38 fg/mL, its manufacturing cost is high due to the difficulty of large-scale production of graphene materials and the involvement of optical systems. Ferreira et al. (2020) prepared an interdigital electrode biosensor [20]. Due to the large size of the interdigital electrode, it is difficult to lower its detection limit, which is 28 ng/mL. With the development of nanomaterials, more and more nanomaterials have been used for the preparation of biosensors [21,22,23]. Among them, silicon nanowires (SiNW) have become an important representative of a new generation of electrochemical sensors due to their unique advantages, such as large specific surface area [24,25], easy surface modification of biological groups [26,27], and easy large-scale manufacturing [28,29]. Because of the compatible processes with CMOS technology, SiNW field effect transistor (FET) sensors have been widely studied [30,31]. For instance, Li et al. (2021) prepared an ultra-sensitive silicon nanowire array biosensor that achieved a low concentration detection of CtDNA at 0.1 fM/L [32,33]. Though the sensitivity of SiNW FET biosensor is well recognized [34], its intrinsic limitation still presents a challenge for continually improving sensitivity and overcoming Debye shielding effect [27,31].

In this paper, in order to improve the sensitivity of the biosensor, we propose to adjust the operating region of the SiNW FET sensor to the subthreshold region and reduce the width of the SiNW. Additionally, the efficiency and stability of the SiNW FET biosensor can be improved by the addition of a PDMS microchannel. Here, we developed a biosensor based on SiNW FET to realize label-free, highly specific, highly sensitive and low-cost detection of Cys-C. Specially, a wafer-level, highly controllable SiNW FET biosensor was fabricated for the quantitative detection of Cys-C. After a series of surface modification processes, Cys-C antibody was assembled on the surface of silicon nanowires to identify and detect Cys-C protein. The detection limit of Cys-C solution using SiNW FET biosensor is as low as 0.43 ag/mL, and the recognition of Cys-C nephrotic markers has high repeatability and specificity.

## 2. Materials and Methods

### 2.1. Materials and Reagents

The experimental reagents were purchased from Beijing InnoChem Technology Co., Ltd., including 3-aminopropyltriethoxysilane (APTES), glutaraldehyde (GA), phosphate-buffered solution (PBS), bovine serum albumin (BSA), and absolute ethanol.

Relevant nephrotic marker proteins and antibodies were provided by Beijing ChunLeiJieChuang Biotechnology Co., Ltd., including rabbit anti human cystatin C polyclonal antibody (anti-Cys-C), recombinant cystatin C (Cys-C), and recombinant human retinol binding protein (RBP).

### 2.2. Fabrication of SiNW FET Biosensor

**Figure 1:**
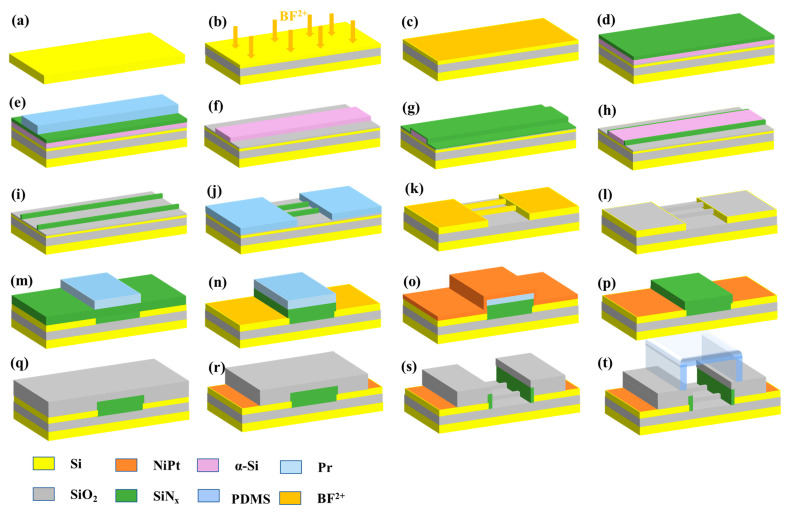
Fabrication flow of SiNW biosensor. (**a**) P-type (100) Si wafers, (**b**) deposition of SiO_2_ and poly-Si films, the surface Si layer is BF^2+^ ion implantation, (**c**) annealing to achieve doping for the implanted ions, (**d**) deposition of multi-layer SiO_2_/α-Si/SiN_x_ films, (**e**) photolithography for gate pattern, (**f**) removal of SiN_x_ to form α-Si arrays, (**g**) deposition of SiNx thin film, (**h**) anisotropic RIE of SiN_x_ to form nanometer SiN_x_ spacers, (**i**) removal of the α-Si material, (**j**) lithography for landing pads, (**k**) dry etching of SiO_2_ and Si, and removal of SiN_x_ and SiO_2_, (**l**) deposition of thin layer SiO_2_, (**m**) deposition of SiN_x_, and photoresist pattern formation of protective channel lithography, (**n**) surface etching of SiN_x_, exposure of the source-drain region, and ion implantation treatment in the source drain region, (**o**) deposition of nickel-platinum (NiPt) alloy, secondary annealing to form metal silicide, (**p**) removal of residual NiPt alloys, (**q**) depositing a thick SiO_2_ layer, (**r**) source leakage holes lithography, etching to remove source drain, (**s**) opening gate graphic lithography and etching gate protection layers SiO_2_ and SiN_x_, (**t**) adding microchannel.

According to our previous research, we prepared wafer level and highly controllable SiNW FET by spacer image transfer (SIT). The top of SiNW was protected by an oxide layer. The SiNW FET biosensors were manufactured on the 8-inch CMOS platform of the Integrated Circuit Advanced Process (ICAC) Research and Development Center, Institute of Microelectronics, Chinese Academy of Sciences (IMECAS). An 8-inch P-type (100) Si wafers with a resistivity of 0.1–100 Ω·cm was selected as the starting material (see Figure 1a). Firstly, the SOI-like structure was prepared by depositing 145 nm SiO_2_ and 40 nm poly-Si, as shown in Figure 1b. To achieve the optimum operating regions of the SiNW Biosensors, different doses of BF^2+^ ions (0, 1 × 10^13^, 5 × 10^13^ and 1 × 10^14^) with 5 keV energy and 7° tilt were implanted, respectively, and ions were activated by spike anneal at 1050 °C (see Figure 1c). Three SiO_2_/amorphous silicon (α-Si)/SiN_x_ films were deposited successively (see Figure 1d). Then, a rectangular array was formed on the surface by the conventional lithography process (see Figure 1e). After dry etching processes of α-Si/SiN_x_ and treatment with H_3_PO_4_ solution at 140 °C, the rectangular α-Si arrays with steep sidewalls were formed (see Figure 1f). In the following step, 30 nm SiN_x_ film was deposited on the surface by plasma enhanced chemical vapor deposition (PECVD), and then the corresponding reactive ion etching (RIE) of SiN_x_ was performed, achieving two SiN_x_ spacers on both sides of the α-Si (see Figure 1g,h). The core α-Si materials were removed by Tetramethylammonium hydroxide (TMAH), leaving only nanometer SiN_x_ spacer arrays (see Figure 1i). In combination with traditional lithography, two landing pads were designed and added to the SiNW array to increase contact area and reduce resistance (see Figure 1j). Subsequently, a dry etch of SiO_2_ and Si were carried out, and the top mask was removed by hot phosphoric acid and diluted hydrofluoric acid (DHF) solutions, respectively (see Figure 1k). The following steps for the formation and preparation of source-drain doping, self-adhesive metal silicide, open-gate grooves and source-drain holes were similar with those described in our previous paper (see Figure 1l–s [35]). Finally, oxygen plasma was, respectively, used to treat the surfaces of the device and the microchannel, and the upper microchannel was bonded to prepare the SiNW biosensor (see Figure 1t).

### 2.3. Surface Modification

The modification of Cys-C probe molecules onto the surface of SiNW requires several steps. First, the surfaces of the sensors were washed repeatedly with ethanol and deionized water. The sensor surface was treated by oxygen plasma for 3 min, and then the microchannel was bonded onto the sensors. Next, a sufficient amount of GA aqueous solution with a concentration of 3% was introduced into the microchannel and soaked for 45 min at room temperature. Then, 1 × PBS solution was selected to clean the microchannel, and then a sufficient concentration of 20 μg/mL of anti-Cys-C solution was introduced, stored in 37 °C incubator for 45 min. Finally, PBS solution was re-introduced into the microchannel for cleaning, and then 0.05% BSA solution was introduced, which was incubated at 4 °C for more than 1 h. After the excess BSA was washed away by introducing PBS solution into the microchannel, a functional layer specifically recognizing Cys-C was formed on the surface of SiNWs, which means that a specific biosensor was prepared to detect Cys-C nephrotic markers. Finally, Cys-C solution was introduced into the pores of PDMS through a syringe and specifically combined with the Cys-C probe.

**Figure 2:**
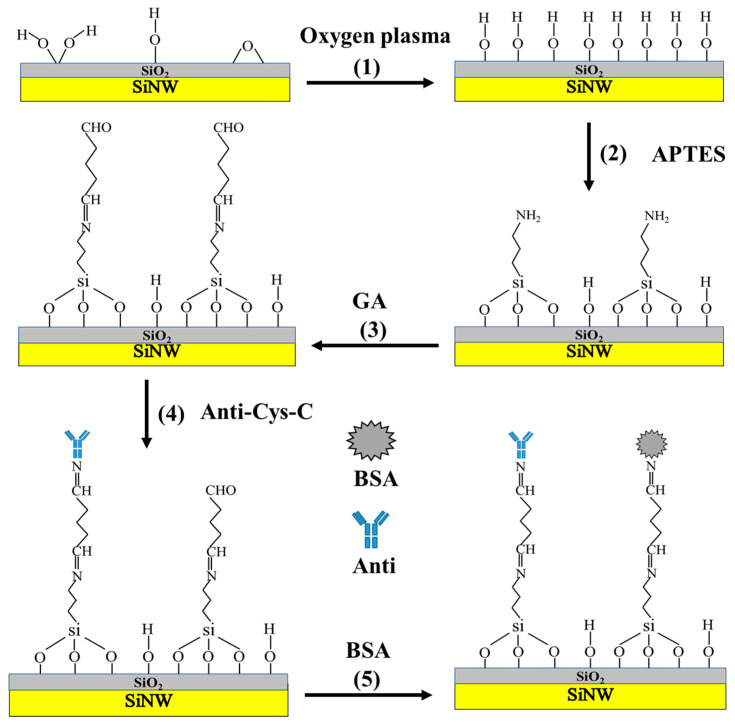
Surface modification flow diagram of SiNW FET biosensor for Cys-C detection.

For biosensors, the sensitivity and stability of biosensors are determined by the functionalization of sensing surfaces by specific molecules. The surface preparation stage based on APTES and GA modification is shown in Figure 2 [36,37]. The key step is to treat the sensor surface with oxygen plasma (30 W, 3 min) to increase the –OH of the surfaces [36], as shown in step (1) in Figure 2. Meanwhile, it can react better with APTES. As shown in step (2), more amino groups are attached to the surface of SiNWs. Then, GA was added, as shown in step (3) of the figure, which can connect with the amino group on the SiNW surface and form aldehyde groups on the SiNW surface. The aldehyde groups on the SiNW surface can connect with the amino group on the protein surface so as to modify the anti-Cys-C on the SiNW surface, as shown in step (4) of Figure 2. Finally, we added a BSA solution and sealed the unmodified groups, as shown in step (5), to avoid non-specific binding.

### 2.4. Working Principles of SiNW FET Biosensors

**Figure 3:**
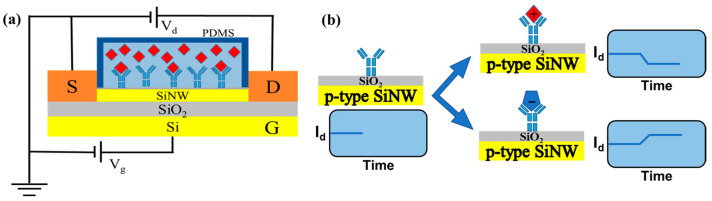
The sensing mechanism of a SiNW FET biosensor. (**a**) Schematic diagram of the SiNW FET biosensor structure, and (**b**) Sensing mechanism of the p-type SiNW FET biosensor.

The sensing mechanism of a SiNW FET biosensor is similar to that of a typical field-effect transistor, as shown in Figure 3a, consisting of a semiconductor channel (SiNW) and a three-electrode system with a source (S), drain (D) and gate (G) electrodes. An external voltage (V_g_) is usually applied to the semiconductor channel between the source and drain, and the resulting current signal is recorded; the gate is responsible for regulating the opening and closing of the semiconductor channel between the source and drain. In the SiNW FET biosensor system, a bioreceptor is immobilized on the surface of the SiNW and recognizes the target analyte by its highly specific binding affinity. When the target is bound by the receptor, the surface potential changes, the channel conductance is modulated, and changes in the internal current of the SiNW are recorded. For example (Figure 3b), when a positively charged target molecule binds to an acceptor molecule immobilized on the surface of a p-type SiNW channel, the hole carriers in the semiconductor channel are reduced, leading to a reduction in the current flowing through the SiNW. Conversely, when negatively charged molecules are trapped by the acceptor, the hole carriers increase, leading to an increase in current. The number of target molecules bound to the surface of the SiNW can determine the magnitude of the internal current. Therefore, the magnitude of the change in current flowing through the SiNW can reflect the concentration of the molecules to be measured.

### 2.5. Characterization of SiNW FET Biosensors

A B1500A (Keysight, Santa Rosa, CA, USA) semiconductor parameter analyzer was used to characterize the electrical properties of the SiNW FET biosensor. The morphology of SiNW sensors was characterized by scanning electron microscope S-4800 (SEM, Hitachi, Tokyo, Japan) and transmission electron microscopy (TEM, FEI Talos, Brno, Czech). Fourier-transform infrared spectroscopy (FTIR)-6300 (FTIR, Jasco Inc., Tokyo, Japan) was used to characterize the modification process.

## 3. Results and Discussion

### 3.1. Structural Characterization and Electrical Characteristics of SiNW FET Biosensor

**Figure 4:**
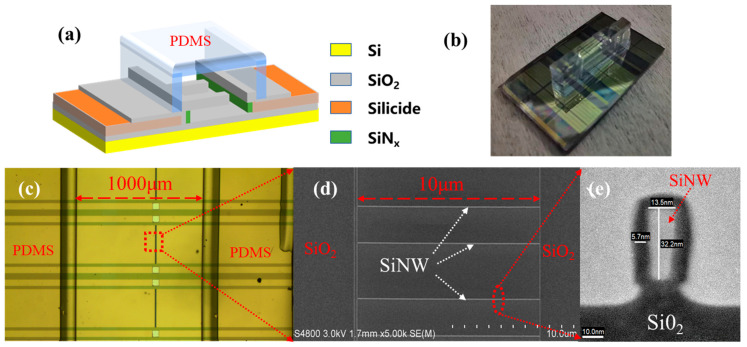
Structure characterization of SiNW biosensor. (**a**) Schematic of the SiNW biosensor with a microfluidic channel, (**b**) physical image of the SiNW biosensor, (**c**) optical microscope image of partially amplified SiNW biosensor, (**d**) SEM image of SiNW array in opened channel, and (**e**) TEM image of SiNW.

The schematics of the SiNW biosensor and the typical images observed during the manufacturing process of the biosensor are shown in Figure 4. Figure 4a shows the schematic of SiNW FET biosensors with a microchannel onto their surfaces. Figure 4b shows the real image of fabricated SiNW FET biosensor with a bonded microchannel, which can lead the antibody molecules and molecules to be measured to the SiNW surface accurately and quickly, contributing to the stability of detection. Figure 4c shows the top view of the SiNW FET biosensor from the microscope, the size of the microchannel with 8 mm in length, 1 mm in width and 100 μm in depth. Figure 4d shows the SEM image of the SiNW arrays. It can be seen from the image that very uniform SiNW arrays were obtained by the SIT approach. Compared with the fabrication approach of SiNW arrays using the traditional electron beam lithography, the SIT technique exhibits the characteristics of high efficiency and low cost. To observe the final profile of SiNW, a cross sectional TEM image is illustrated in Figure 4e. The SiNW width has been reduced to 13.5 nm and the height to 32.2 nm by a thin film thinning process. Compared with the previous work, the size of the SiNW array is well controlled and much smaller, which has greatly increased the specific surface area of the SiNW and is helpful to achieve a higher sensing sensitivity [38].

**Figure 5:**
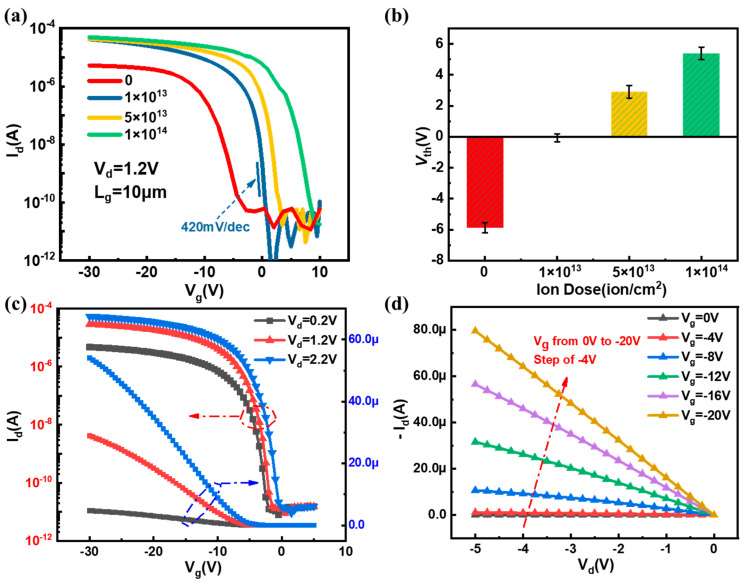
Characterization of electrical properties of SiNW biosensor. (**a**) Transfer curves of SiNW biosensor with different implanted doses, (**b**) statistical values of *V*_th_s of SiNW biosensor with different implanted doses, the transfer (**c**) and output (**d**) curves of the SiNW biosensor with the BF^2+^ doses of 1 × 10^13^ (The dotted frame in figure (**c**) correspond to the different coordinate axis scales.).

Figure 5 shows the electrical characteristics of SiNW FET biosensor. It can be seen from Figure 5a that the transfer curves of SiNW FET biosensor gradually shifts to the right with the increase in doses of implanted BF^2+^ ions, and thus the threshold voltages (*V*_th_) changed to a positive direction. The *V*_th_ of SiNW FET biosensors with different doses was extracted according to the constant current method. As shown in Figure 5b, with the increase in doping doses, the values of *V*_th_ shift to the positive direction. The minimum SS (420 mV/dec) is achieved with the implantation of BF^2+^ ions of 1 × 10^13^, and the *V*_th_ in the SiNW biosensor is approximately 0 V. Considering the mechanism of SiNW FET sensors, the sensor would have an exponential change in sensitivity when the device operates within the threshold voltage range [39]. Therefore, the SiNW FET biosensor with implantation of 1 × 10^13^ was selected to detect the early kidney failure biomarker Cys-C. The transfer and output curves of the optimized SiNW FET biosensor are shown in Figure 5c,d, respectively. With the increase in negative gate voltages, the current of the SiNW biosensor also increases, and the I_on_/I_off_ ratio is over 10^6^, exhibiting good electrical characteristics. The above results reveal that the gate of SiNW FET sensor has a good regulating effect on the current and can be used for the manufacturing and detection of biosensors.

### 3.2. Surface Modification

**Figure 6:**
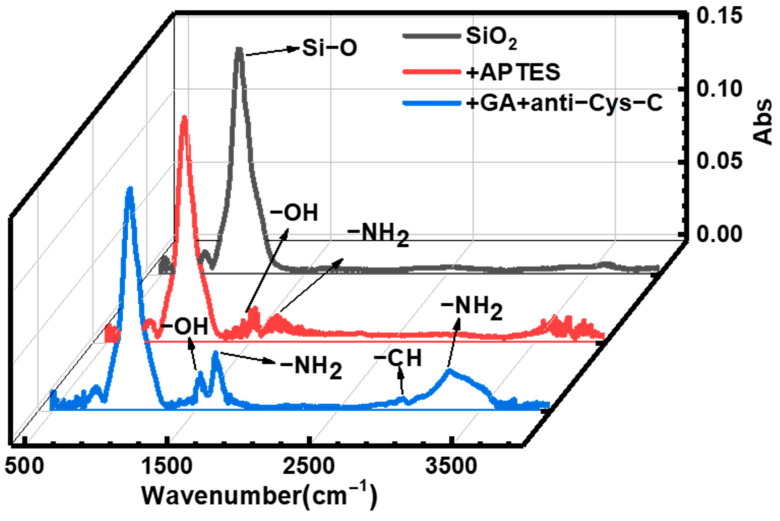
FTIR characterization of anti-Cys-C modified on SiNW surface.

The infrared absorption peak of SiO_2_ surface during the modification process was analyzed by infrared spectrometer, and the change of the absorption peak fully reflected the change of SiNW surface groups during the modification processes, as shown in Figure 6. After soaking in APTES, it can be seen from the spectrum that there are obvious hydroxyl and amino groups on the surface of SiO_2_, which is due to the treatment of oxygen plasma and the presence of three amino groups in APTES. After being treated with GA and Anti-Cys-C, more amino groups and methyl groups appeared in the spectrum. This is consistent with the antibody protein modified on the surface of SiNW. Therefore, the change of FTIR spectrum indicates that the silane method can modify the anti-Cys-C on the sensor layer on the SiNW surface.

### 3.3. Detection of Cys-C and Sensitivity

**Figure 7:**
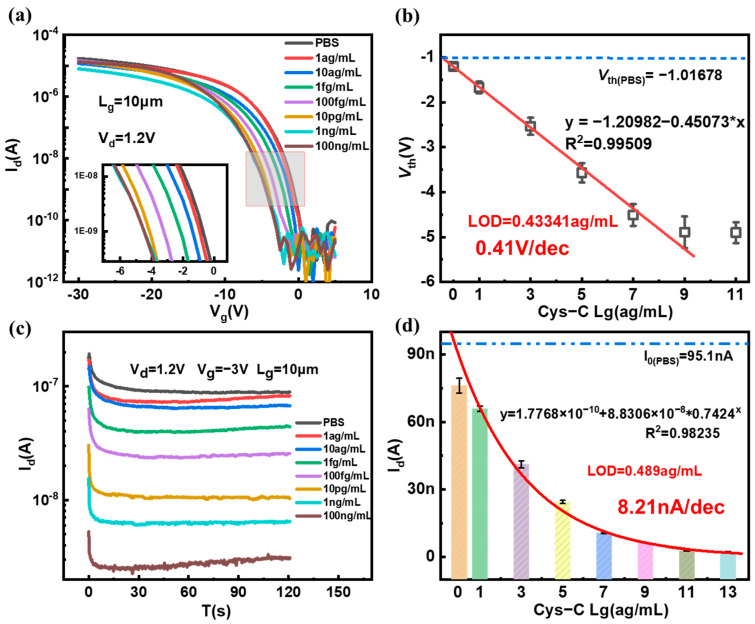
Electrical response of SiNW FET biosensor to different concentrations of Cys-C. (**a**) Transfer curves of the SiNW FET biosensor interacting with varying concentrations of Cys-C (The red frame indicates the change in the curve around the sub-threshold region and is shown enlarged.), (**b**) the *V*_th_s of the SiNW FET biosensor in a series of Cys-C concentrations, (**c**) real-time response of Cys-C solution with different concentrations, and (**d**) the relationship between the steady-state drain current and the concentration of Cys-C.

To achieve the sensitivity of the SiNW FET biosensor for Cys-C detection, the samples with different concentrations (range from 1 ag/mL to 10 μg/mL.) were introduced to the surfaces of the SiNW FET biosensor with the modified anti-Cys-C, and the I_d_-V_g_ curves and real-time current changes of the SiNW FET biosensor were recorded. Specifically, the B1500A semiconductor parameter analyzer was used to record the signal variation of the SiNW FET biosensor after the Cys-C solution was introduced into the microchannel of the sensor. As the positive Cys-C protein molecules specifically bonded to the probe molecules on the surface of SiNW, the concentration of carriers (holes) in the SiNW was reduced, resulting in an increase in SiNW resistance. Meanwhile, the current flowing through SiNW would decrease accordingly, and the transfer characteristic curve would shift to the left simultaneously. Furthermore, with the increase in the concentration of Cys-C solution, more Cys-C protein molecules would be captured on the SiNW surface, which would result in the gradual left shift of the transfer curves and the decrease in the current flowing through SiNW. The electrical response of SiNW FET biosensor to different concentrations of Cys-C is shown in Figure 7a,c.

The *V*_th_s is extracted according to the transfer curves with different concentrations of Cys-C. The *V*_th_ has a linear relationship with the logarithm of the Cys-C concentration. Figure 7b shows the relationship between the *V*_th_ and the Cys-C concentration. Obviously, there is a perfect linear relationship between 1 ag/mL and 1 ng/mL, the linear regression equation after calibration and fitting is y = −1.20982 − 0.45073 ∗ x and R^2^ = 0.999509, where x is the logarithm of Cys-C concentration, and the detection limit of Cys-C was obtained through linear fitting, with LOD = 0.43341 ag/mL. The average sensitivity to *V*_th_ change is 0.41 V/dec. We took the average value and standard deviation of the stable current in the real-time current curve and drew the relationship shown in Figure 7d. It was found that the average current of the real-time current has an exponential relationship with the logarithm of the Cys-C concentration, especially in the concentration range of 1 ag/mL~10 μg/mL. The regression equation after calibration and fitting was y = 1.7768 × 10^−10^ + 8.8306 × 10^−8^ ∗ 0.7424^x^ and R^2^ = 0.98235, where x is the logarithm of Cys-C concentration. The detection limit current was obtained by subtracting three times the standard deviation from the average current of the PBS group, and the detection limit was obtained by fitting with LOD = 0.489 ag/mL.

The ultrasensitive detection achieved by the SiNW biosensor prepared in this paper may be related to the optimization we have made to the sensor device. Firstly, the operating region of the SiNW FET biosensor was modulated to the subthreshold region by doping SiNW with BF^2+^ ions because the highest sensitivity detection can be achieved in the subthreshold region. Next, increasing the specific surface area of SiNW can improve the detection sensitivity of SiNW FET biosensors. In this study, SiNW, with a width of about 13 nm and a height of about 32 nm, was prepared by a process thinning technique to achieve an increase in the specific surface area of SiNW. Finally, the thinner the sensing layer of the biosensor is, the more charged proteins are bounded to the surface of the SiNW, which can better influence the carrier distribution inside the SiNW and improve the detection sensitivity. The sensing layer thickness of the biosensor designed in this study was 6 nm, which could realize more sensitive detection. In summary, by optimizing the preparation process of the SiNW FET biosensor, the biosensor prepared in this study realized the ultrasensitive detection of Cys-C.

### 3.4. Repeatability and Specificity of SiNW FET Biosensor

**Figure 8:**
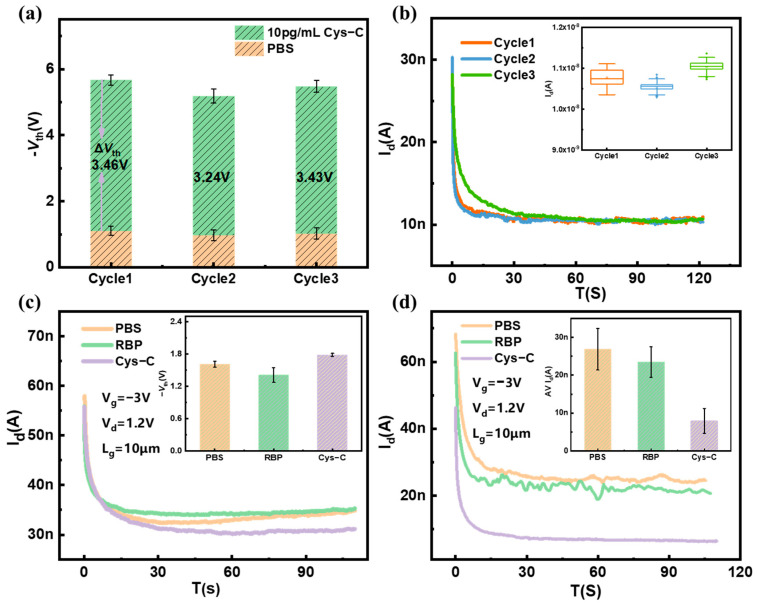
Specificity and repeatability of the SiNW FET biosensor. The normalized electrical response of 10 pg/mL Cys-C on (**a**) different SiNW FET biosensors, (**b**) the same SiNW FET biosensors, (**c**) the electrical response of the unmodified antibody SiNW FET biosensor to different early kidney failure biomarkers, and (**d**) the electrical response of the modified antibody SiNW FET biosensor to different early kidney failure biomarkers.

Repeatability is a very important factor for biosensors, and the repeatable performance results of the sensor are shown in Figure 8a. The *V*_th_s of the three biosensors for detection of 10 pg/mL Cys-C are 3.46 V, 3.26 V, and 3.43 V, respectively, exhibiting good repeatability. The small errors in the experimental results may be related to the uncertainty in the number of antibodies modified on the SiNW surface. Then, the repeatability was studied using the same device for multiple real-time detections of the 10 pg/mL Cys-C, as shown in Figure 8b. It is clear that the real-time current curve flowing through the sensor shows a high degree of repeatability, and the statistical distribution of the current taken in the figure is stable.

To verify the specific recognition of Cys-C using the SiNW FET biosensor, different early kidney failure biomarkers (Cys-C and RBP) with the same concentration (1 ng/mL) were introduced into the SiNW FET biosensor. For the surface of SiNW modified without any antibody, the current of SiNW FET biosensor does not change significantly after the addition of nephrotic markers, the current levels are all around 35 nA, as shown in Figure 8c. Consequently, the change of *V*_th_s is rarely small, as is shown in the inserted figure. Then, RBP and Cys-C solutions with same concentration (1 ng/mL) were introduced into the microchannel of SiNW FET biosensor modified with anti-Cys-C, respectively. Since the surface of SiNW was modified with anti-Cys-C, there is no obvious change in current of the SiNW biosensor after introducing the RBP solution. However, the current of the SiNW FET biosensor decreased significantly when Cys-C solution was introduced; the current dropped from 27 nA to 8 nA, as shown in Figure 8d. The results demonstrate that the SiNW FET biosensor has good specificity to detect renal markers.

So far, biosensors have rarely been used to detect Cys-C. Although they provide a low detection range and low detection sensitivity (Table 1), there exist some limitations in medical care point diagnosis. Compared with other detection methods, we can get a lower detection limit by using SiNW FET biosensor, which is inseparable from the larger surface-to-volume values and modified suitable operating region. Furthermore, from the perspective of preparation materials, the SiNW FET biosensor is more likely to be prepared on a large scale because it is compatible with the COMS process. Therefore, our ultrasensitive detection method shows great advantages in LOD and preparation. Point-of-care testing (POCT) is a way of performing diagnostic tests outside the laboratory to produce fast and reliable results to help identify disease. SiNW FET biosensors are well suited to the needs of point-of-care testing due to them being fast, high sensitivity and label-free. Additionally, because the preparation process is compatible with CMOS processes, SiNW FET biosensors have the advantages of easy miniaturization and low-cost, large-scale fabrication. Considering all these aspects, SiNW FET biosensors have outstanding advantages in POCT.

## 4. Conclusions

In this paper, a new method for the quantitative detection of the Cys-C early kidney failure biomarker has been proposed by using a SiNW FET biosensor. This method is label-free, fast and ultrasensitive. By doping SiNWs, the *V*_th_ of the SiNW FET biosensor was regulated, the SS of the device was effectively reduced, and the detection sensitivity of the sensor was also improved. In addition, the anti-Cys-C was modified on the SiO_2_ layer on the surface of SiNW using the silylation method to ensure that the sensor only specifically captures the specific Cys-C protein in the solution. The experimental results show that the prepared SiNW FET biosensor has obvious advantages in the detection of the early kidney failure biomarker Cys-C. When the concentration of Cys-C ranges from 1 ag/mL to 1 ng/mL, linear detection can be carried out, and the detection limit fitted is 0.43 ag/mL. The SiNW biosensor can monitor early kidney failure biomarkers with ultra-high sensitivity, which has a high potential in future clinical medical detection.

## Figures and Tables

**Table 1:** Biosensors and immunosensors for detection of Cys-C.

Method	Material	Linear Range and (LOD)	Reference
Differential Pulse Voltammetry (DPV)	Au@Fe_3_O_4_	0.01 pg/mL~30 ng/mL (3 fg/mL)	Yang et al., 2016 [16]
Photocurrent response	TiO_2_ nanotubes	0.72 pM~3.6 nM (0.14 pM)	Mi et al., 2016 [17]
Square wave voltammetry (SWV)	Prepared poly(thionine)-Au	100 ng/mL~10 fg/mL (4.6 fg/mL)	Wang et al., 2017 [40]
Cyclic voltammetry and differential pulse voltammetry	Multiwalled carbon nanotube (MWCNT)	0.6~6.6 ng/mL (0.58 pg/mL)	Desai et al., 2018 [18]
Linear sweep voltammetry (LSV)	AuNPs	10~100 ng/mL (6.0 ng/mL)	Lopes et al., 2019 [15]
Square wave voltammetry (SWV)	Graphene oxide-ferrocene nanofilm	0.1~1000 ng/m L (0.03 ng/mL)	Erika et al., 2019 [41]
Eletrochemiluminescent (ECL)	Graphene composite(G/mRub)	1.0 fg/mL~10 ng/mL (0.38 fg/mL)	Zhao et al., 2019 [19]
Interdigitated electrode (IDE)	Polypyrrole/carbon nanotube	0~300 ng/mL (28 ng/mL)	Ferreira et al., 2020 [20]
Field Effect Transistor (FET)	Silicon nanowire	1 ag/mL~1 ng/mL (0.43341 ag/mL)	This work

## Data Availability

Not applicable.
