# Correction: Hu et al. Ultrasensitive Silicon Nanowire Biosensor with Modulated Threshold Voltages and Ultra-Small Diameter for Early Kidney Failure Biomarker Cystatin C. *Biosensors* 2023, *13*, 645

**DOI:** 10.3390/bios14100461

**Published:** 2024-09-26

**Authors:** Jiawei Hu, Yinglu Li, Xufang Zhang, Yanrong Wang, Jing Zhang, Jiang Yan, Junjie Li, Zhaohao Zhang, Huaxiang Yin, Qianhui Wei, Qifeng Jiang, Shuhua Wei, Qingzhu Zhang

**Affiliations:** 1 School of Information Science and Technology, North China University of Technology, Beijing 100144, China; hujiawei@ime.ac.cn (J.H.); liyinglu@ime.ac.cn (Y.L.); zhangxufang@ncut.edu.cn (X.Z.); wangyanrong@ncut.edu.cn (Y.W.); zhangj@ncut.edu.cn (J.Z.);; 2 Advanced Integrated Circuits R&D Center, Institute of Microelectronic of the Chinese Academy of Sciences, Beijing 100029, China; lijunjie@ime.ac.cn (J.L.); zhangzhaohao@ime.ac.cn (Z.Z.); yinhuaxiang@ime.ac.cn (H.Y.); 3 State Key Laboratory of Advanced Materials for Smart Sensing, General Research Institute for Nonferrous Metals, Beijing 101402, China; weiqianhui@grinm.com

## 1. Errors in Figure and Table

In the original publication [1], there was a mistake in Figure 7b as published. Figure 7b contained a human error. When creating the figure, due to the similarity with the other figures, we chose the wrong one by mistake. The correct version of Figure 7 appears below.

Correspondingly, there was a mistake in Table 1. The LOD of this work should be 0.2529 ag/mL. The correct version of Table 1 appears below.

## 2. Text Correction

There were some textual errors related to Figure 7b in the original publication. 

There was an error in the Abstract Section, “in the range of Cys-C concentration from 1 ag/mL to 10 pg/mL”, in which “10 pg/mL” is wrong.

A correction has been made to the Abstract Section as follows:

“in the range of Cys-C concentration from 1 ag/mL to 1 ng/mL”.

There was an error in the Introduction Section, “The detection limit of Cys-C solution using SiNW FET biosensor is as low as 0.43 ag/mL”, in which “0.43 ag/mL” is wrong.

A correction has been made to the Introduction Section, paragraph 4, as follows:

“The detection limit of Cys-C solution using SiNW FET biosensor is as low as 0.25 ag/mL”.

There was an error in the Results and Discussion Section, “the linear regression equation after calibration and fitting is y = −1.20982 − 0.45073 × x and R^2^ = 0.999509, where x is the logarithm of Cys-C concentration, and the detection limit of Cys-C was obtained through linear fitting, with LOD = 0.43341 ag/mL. The average sensitivity to V_th_ change is 0.41 V/dec.”, in which “y = −1.20982 − 0.45073 × x and R^2^ = 0.999509”, “LOD = 0.43341 ag/mL” and “V_th_ change is 0.41 V/dec” is wrong.

A correction has been made to Results and Discussion Section, subsection 3.3, Detection of Cys-C and Sensitivity, paragraph 2, as follows:

“the linear regression equation after calibration and fitting is y = −1.27318 − 0.42954 × x, R^2^ = 0.98734, where x is the logarithm of Cys-C concentration, and the detection limit of Cys-C was obtained through linear fitting, with LOD = 0.2529 ag/mL. The average sensitivity to V_th_ change is 0.42 V/dec”.

There was an error in the Conclusions Section, “the detection limit fitted is 0.43 ag/mL”, in which “0.43 ag/mL” is wrong.

A correction has been made to the Conclusions Section as follows:

“the detection limit fitted is 0.25 ag/mL”.

The authors apologize for any inconvenience caused and state that the scientific conclusions are unaffected. This correction was approved by the Academic Editor. The original publication has also been updated.

## Figures and Tables

**Figure 7:**
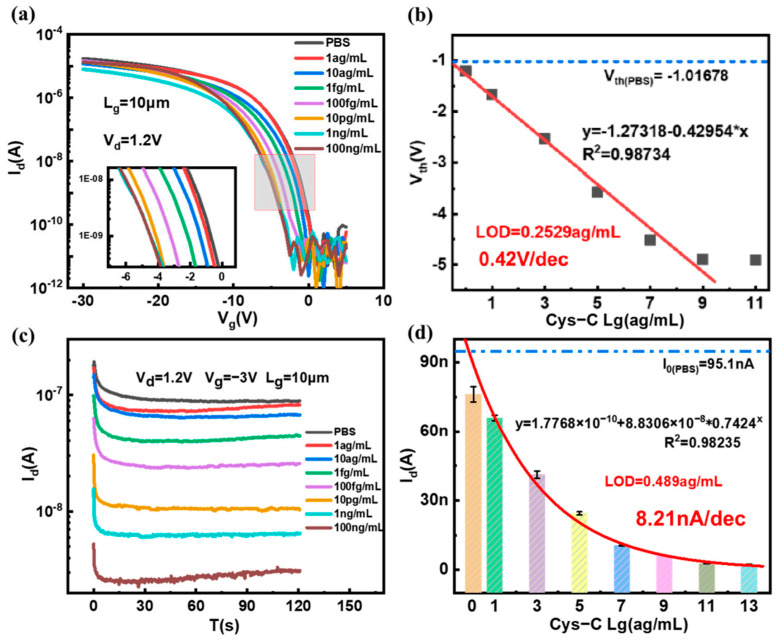
The electrical response of the SiNW FET biosensor to different concentrations of Cys-C. (**a**) Transfer curves of the SiNW FET biosensor interacting with varying concentrations of Cys-C (the red frame indicates the change in the curve around the sub-threshold region and is shown enlarged), (**b**) the V_th_s of the SiNW FET biosensor in a series of Cys-C concentrations, (**c**) the real−time response of the Cys-C solution with different concentrations, and (**d**) the relationship between the steady−state drain current and the concentration of Cys-C.

**Table 1:** Biosensors and immunosensors for detection of Cys-C.

Method	Material	Linear Range (LOD)	Reference
Differential pulse Voltammetry (DPV)	Au@Fe_3_O_4_	0.01 pg/mL~30 ng/mL (3 fg/mL)	Yang et al., 2016 [16]
Photocurrent response	TiO_2_ nanotubes	0.72 pM~3.6 nM (0.14 pM)	Mi et al., 2016 [17]
Square wave voltammetry (SWV)	Prepared poly(thionine)-Au	100 ng/mL~10 fg/mL (4.6 fg/mL)	Wang et al., 2017 [40]
Cyclic voltammetry and differential pulse voltammetry	Multiwalled carbon nanotube (MWCNT)	0.6~6.6 ng/mL (0.58 pg/mL)	Desai et al., 2018 [18]
Linear sweep voltammetry (LSV)	AuNPs	10~100 ng/mL (6.0 ng/mL)	Lopes et al., 2019 [15]
Square wave voltammetry (SWV)	Graphene oxide-ferrocene nanofilm	0.1~1000 ng/mL (0.03 ng/mL)	Erika et al., 2019 [41]
Electrochemiluminescent (ECL)	Graphene composite (G/mRub)	1.0 fg/mL~10 ng/mL (0.38 fg/mL)	Zhao et al., 2019 [19]
Interdigitated electrode (IDE)	Polypyrrole/carbon nanotube	0~300 ng/mL (28 ng/mL)	Ferreira et al., 2020 [20]
Field effect transistor (FET)	Silicon nanowire	1 ag/mL~1 ng/mL (0.2529 ag/mL)	This work
